# Healthcare-Associated Infections (HAIs): Challenges and Measures Taken by the Radiology Department to Control Infection Transmission

**DOI:** 10.3390/vaccines10122060

**Published:** 2022-11-30

**Authors:** Ali Alamer, Fawaz Alharbi, Asim Aldhilan, Ziyad Almushayti, Khalefa Alghofaily, Ayman Elbehiry, Adil Abalkhail

**Affiliations:** 1 Department of Radiology, College of Medicine, Qassim University, Buraydah 51452, Saudi Arabia; 2 Department of Public Health, College of Public Health and Health Informatics, Qassim University, Buraydah 52741, Saudi Arabia

**Keywords:** healthcare-associated infections (HAIs), radiology department, imaging, radiology equipment, infection prevention and control, infection surveillance, healthcare workers

## Abstract

Infections contracted during healthcare delivery in a hospital or ambulatory setting are collectively referred to as healthcare-associated infections (HAIs). Healthcare workers and patients alike are vulnerable to serious problems as a result of the risk of HAIs. In the healthcare system, HAIs are considered among the most common and serious health problems. However, the occurrence of HAIs differs between different types of clinical departments within the hospital. Recently, the risk of HAIs has been increasing in radiology departments globally due to the central role of radiology in guiding clinical decisions for the diagnosis, treatment, and monitoring of different diseases from almost all medical specialties. The radiology department is particularly vulnerable to HAIs because it serves as a transit hub for infected patients, non-infected patients, and healthcare workers. Furthermore, as the number of patients referred to radiology and the length of patient contact time has increased, thanks to modern imaging techniques such as computed tomography and magnetic resonance imaging, the risk of HAIs has also increased significantly. With the increasing use of interventional radiological procedures, patients and healthcare workers face a potentially greater risk of contracting HAIs due to the invasive nature of such procedures. Although not exhaustive, we attempted through a literature search to provide a general overview of infection prevention and control practices, address HAIs in the radiology departments, and highlight the challenges and measures taken to control infection transmission in the radiology departments.

## 1. Introduction

Healthcare-associated infections (HAIs), nosocomial infections, and hospital infections are contagious illnesses that manifest two days after admission to the hospital, within three days of discharge, or 30 days after receiving medical attention [1,2]. Infections can be acquired anywhere in a healthcare setting, including long-term units, personal care, or outpatient clinics [3]. HAIs can also refer to epidemics associated with the utilization of instruments or medical procedures and practices [4], such as the carbapenem-resistant Enterobacteriaceae infections linked to the use of duodenoscopes (used mostly for endoscopic cholangiopancreatography) despite strict adherence to manufacturer guidelines and industry standards [5]. HAIs pose significant challenge for both healthcare workers and patients [6]. From 2016 to 2017, point prevalence surveys of HAIs and antimicrobial usage in the European Union and European Economic Area (EU/EEA) included 310,755 patients from acute care hospitals and 117,138 residents from long-term care facilities across various countries. According to country-weighted prevalence estimates, 6.5% of acute care hospital patients and 3.9% of long-term care facility residents had at least one HAI (cCI: 5.4–7.8%) [7]. In underdeveloped nations, the danger of catching HAIs is twenty times greater than in developed nations [8]. This correlates to a maximum rate of infection of 25% [9]. HAIs result in higher rates of death and morbidity, longer hospital admissions, antimicrobial resistance, and higher healthcare expenses [10,11,12]. For example, in the United States (US), HAIs cause between 44,000 and 98,000 unexpected deaths and the cost of dealing with these infections is estimated at US $17–29 billion [13]. According to national and multicenter data in the US, the rate of HAIs is 6.6% [14]. According to a report by the World Health Organization (WHO) on the burden of HAIs around the world, the rate of HAIs ranges from 3.6 to 12% in high-income countries and from 5.4 to 19.1% in low- and middle-income countries [15].

The rate of HAIs may vary amongst clinical departments within the same institute depending on the nature of the clinical services that are offered. Because of the essential role that the radiology department plays in the diagnosis and management of various disorders across practically all medical disciplines, patients and healthcare workers alike are at risk of contracting HAIs. In fact, the radiology department was labeled as a “high-risk area” for HAIs in some reports [16,17]. According to Ustünsöz [16], the risk of HAIs in radiology departments might be further exacerbated by a lack of necessary knowledge among radiology personnel, as well as a lack of standards to control infection transmission. Therefore, in this review, we will discuss the challenges and measures taken to control infection transmission particularly in radiology departments.

## 2. Methods

A literature search was performed in June 2022 using PubMed for “healthcare-associated infections”, “HAIs”, “radiology department”, “imaging”, “interventional radiology”, “radiology equipment”, “infection prevention and control”, “infection surveillance”, and “healthcare workers”. Boolean operators, truncation, nesting for synonyms, and quotation for phrases were used to refine our search to most relevant articles. Two authors (Alamer and Abalkhail) screened the abstracts of the articles for relevance. Although not exhaustive, the search includes important and relevant articles that address the challenges and measures taken to control infection transmission particularly in radiology departments. To augment this review, references cited within the identified articles were also used.

To achieve its aim, this review was divided into two major parts. The first part attempts to provide a general review of current infection prevention and control practices for healthcare workers. It consists of the following sections: an overview of diseases and organisms in healthcare settings—HAIs, infection prevention and control strategies and challenges, and infection surveillance systems. The second part tries to review infection prevention and control practices that are more relevant to radiology departments. It consists of the following sections: HAIs in radiology departments, challenges of infection prevention and control in radiology departments, and measures taken by the radiology departments to control infection transmission.

## 3. An Overview of Diseases and Organisms in Healthcare Settings—HAIs

A point-prevalence survey was conducted between September and December 2016 in participating hospitals following guidelines from the European Centre for Disease Prevention and Control [18]. Ten institutions, or 32.9% of all acute care beds in government hospitals, were examined. There were 184 HAIs in 172 patients, with an overall prevalence of 8.2%. Rates of infection in hospitals ranged from 3.5% to 14.4%, with higher infection rates in secondary and tertiary care facilities. Respiratory infections (16.3%), urinary infections (18.5%), bloodstream infections (19.5%), and surgical site infections (32.6%) were the most common HAIs. Seven percent of HAIs were caused by infections connected to devices. *Escherichia coli* (*E. coli*) was the most frequently isolated microorganism and was found in 12.5% of HAIs. Sixty-one percent of all patients polled were taking antibiotics, and 89% of those with HAIs had received at least one antimicrobial medication as of the survey date.

The Centers for Disease Control and Prevention (CDC) classifies 50 nosocomial infection sites into 13 categories according to biological and clinical standards [19]. Urinary infections, surgical and soft tissue infections, skin infections, bacteremia, gastroenteritis, meningitis, and respiratory infections are examples of common infections [20]. *Staphylococcus aureus* (*S. aureus*), *Pseudomonas aeruginosa* (*P. aeruginosa*), and *E. coli* are the bacteria most frequently responsible for HAIs. Although, bacteria are not the main source of HAIs [21]. Fungi, such as *Candida albicans* and *Aspergillus* spp., and viruses, such as the respiratory syncytial virus and influenza virus, can also cause HAIs. HAIs also include Methicillin-resistant *Staphylococcus aureus* (MRSA), Methicillin-sensitive *Staphylococcus aureus* (MSSA), vancomycin-resistant *Enterococci* (VRE), and multidrug-resistant *Acinetobacter* spp. [22].

In the Kingdom of Saudi Arabia (KSA), few studies have examined the prevalence of different types of HAIs. According to Sabra and Abdel-Fattah [23] who conducted a study of HAIs in Taif Hospitals in KSA, 32.3% of HAIs cases were respiratory tract infections (RTI), 25.3% urinary tract infections (UTI), 18.2% were bloodstream infections (BI), and 12.9% were surgical site infections (SSI). Among the biological agents that were identified, MRSA, coagulase-negative *Staphylococci* (CNS), and *S. aureus* accounted for the majority (31.7%) of the gram-positive isolates. *E. coli* 22.3%, *P. aeruginosa* 17.6%, and *Klebsiella pneumoniae* (*K. pneumoniae*) 9.9% made up the majority of the gram-negative isolates (66.3%). Two percent of all isolates were *Candida* spp. The most typical isolate from urinary infections was *E. coli* (47.7%), followed by *K. pneumoniae* (15.1%), and *P. aeruginosa* (8.2%). *Acinetobacter* spp. (12%), MRSA (14.8%), and *P. aeruginosa* (44.4% of respiratory isolates) were found. *K. pneumoniae*, CNS, and *E. coli* were the three most common isolates from bloodstream infections. *E. coli* 25.6%, MRSA 18.6%, and MSSA 14% were the most common SSI isolates.

## 4. HAIs in Radiology Departments

In radiology departments, the risk of HAIs is on the rise due to the increasing number of patients and extending the role of medical imaging to include more interventions [24]. In fact, radiology departments have undergone substantial revolutionary changes throughout the last few decades with dramatic rise in numbers of diagnostic medical imaging. Such changes were linked to emerging cross-sectional imaging applications such as computed tomography (CT) and magnetic resonance imaging (MRI), which expanded radiology’s central role in patients’ care [25]. It is estimated that around 88 million CT scans and 42 million MRI scans are performed annually in the US alone [26]. As well as increasing the number of patients, using these new and complex imaging techniques has increased contact time between patients and healthcare workers in radiology departments which also has increased the risk of contracting HAIs [16].

Furthermore, interventional radiological applications have added more responsibility and a potentially higher risk for HAIs as a result of the invasive nature of procedures and direct patient contact [16,27,28]. The use of percutaneous techniques for diagnostic and therapeutic endovascular interventional radiology is on the rise, and with that comes the possibility of an increase in adverse effects including HAIs [29]. Every year, over seven million patients all around the world undergo percutaneous interventional vascular procedures, and it is anticipated that this number will continue to rise in the years to come [30]. Despite that, there have only been a few studies that have attempted to evaluate the incidence of HAIs following radiological interventional procedures. The data were diverse and variable, and they were likely affected by a wide variety of factors, such as the monocenteric basis of the study and the type of procedure. For instance, due to the fact that biliary drainage can be categorized as “clean/contaminated” procedure, the risk of infectious complications following transhepatic cholangiogram is likely the highest of any interventional procedure, at up to 40% [31,32]. On the other hand, the overall incidence of procedure-related infections is only 0.1% following the less invasive ultrasound-guided procedures such as ultrasound-guided biopsy and fine-needle aspiration [33].

During the Coronavirus disease 2019 (COVID-19) pandemic, radiology departments had a key role in managing the pandemic, diagnosing patients and identifying those likely to need intensive care [34]. The use of medical imaging is crucial in guiding clinical decisions for the diagnosis, treatment, and monitoring of patients with COVID-19 [35,36]. During the COVID-19 pandemic, the radiology department was considered a “high-risk area” because it served as a transit hub for infected patients, non-infected patients, and healthcare workers [17]. Infections can be transmitted from infected patients through direct contact, indirect contact, droplet and/or airborne routes. Infections can be contracted in common waiting areas, examination rooms, procedure holding areas, and procedure rooms. Therefore, the risk of infection exposure is not confined to radiologists, technologists, and nurses, but also affects receptionists, transport staff, monitoring personnel, and patients [37]. Göhler et al. [38], reported considerable contamination on the surface of the CT scanner after examination of different COVID patients, despite the fact that no viral cell culture positive was found, implying that the infectious potential was minimal. Therefore, it is of utmost importance for radiology departments to establish clear measures to control infection transmission [24].

## 5. Infection Prevention and Control Strategies and Challenges

Depending on the route of transmission of the infectious agent, transmission-based precautions (TBPs) are commonly categorised into contact, droplets, airborne, enteric, and bloodborne precautions [24,39,40,41,42,43,44]. Table 1 summarized the most frequent forms of HAIs, their modes of transmission, and examples of precautionary measures.

In the healthcare environment, there are multiple infection prevention and control (IPC) strategies with specific protocols that healthcare workers must follow. Lack of adherence to these protocols could be due to a lack of training, lack of perceived risk, or lack of interest in IPC amongst healthcare workers [45]. Therefore, it is important to examine the factors that determine the successful implementation of new IPC strategies. Training and education of all new and existing staff, regardless of their position, can be helpful to make sure that staff are well informed about new IPC methods [46]. The success of implementing IPC strategy, at least in its initial stages, relies to a great extent on staff training and supervision [45]. Moreover, increasing the knowledge of IPC practices among healthcare workers is crucial for reducing the transmission of infections [45]. Based on a literature review carried out by Moralejo et al. [47], who assessed adherence to IPC practices among individuals and organisations, significant variations were observed in baseline adherence to IPC and the extent of changes both between and within studies as well as by the specific behaviour assessed. They concluded that because IPC practices can play a key role in minimizing transmission, it is logical for organizations to analyse adherence and contributing factors locally, as well as develop, execute, and evaluate interventions tailored to their needs.

## 6. Challenges of Infection Prevention and Control in Radiology Departments

Patients and healthcare workers within the radiology department are susceptible to HAIs because of the department’s central role in the identification and management of numerous ill patients [24,48]. Because of the high cost and lack of portability of radiology equipment, patients from various medical services are referred to a single location for diagnostic imaging and therapeutic interventions [16]. Due to the fact that such a practice requires providing radiology services in a single location to a diverse range of patients, including emergency patients, inpatients, and outpatients, the radiology department is considered as a “higher risk area” in term of HAIs [17].

Radiology equipment contamination such as X-ray or CT machines is one of the major concerns associated with HAIs in radiology departments [38,49]. The reported contamination in the literature was not limited to radiology machines and includes radiation personal protective equipment (PPE) and radiology workstations [50,51]. Furthermore, the interventional suite, where there is direct patient contact and invasive procedures are performed, presents another infection control challenge for the radiology department [29]. Outside of the examination rooms, additional sites in radiology departments where HAIs might be contracted include reception desks, halls, and waiting areas [24]. The waiting areas in the radiology departments of most hospitals have limited space, and the areas can get quite crowded, which raises the risk of contracting HAIs [52]. Therefore, the healthcare workers in radiology departments, such as physicians, technologists, and nurses, should have up-to-date knowledge of IPC practices to diminish the spread of HAIs via radiology equipment or personnel [24,53].

Unfortunately, the literature demonstrated that healthcare personnel in the radiology department, including radiologists, lacked an up-to-date knowledge of IPC practices [37]. Reddy et al. [54], assessed the IPC practices among interventional radiologists, and they found only 44% of the respondents had infection control training prior to starting practice. They also found that only 19% of the surveyed interventional radiologists routinely washed their hands between glove applications, and that slightly more than half of the participants wore gowns, caps, and used full barrier precautions. Another study that surveyed diagnostic radiologists and radiology residents found that 54% of the participants had never attended an IPC training session [55]. In addition, the insufficient knowledge of IPC practices extended beyond radiologists to encompass other radiology personnel, as evidenced by the absence of specialized training sessions in the professional education of technologists [16].

## 7. Infection Surveillance Systems

To effectively plan, implement, and evaluate healthcare practices and systems in a timely and consistent manner, to ensure that the data is made available to those who need it, surveillance is required. Surveillance in this context is defined as the continuous, systematic collection, analysis, and interpretation of healthcare data [56]. Based on WHO, surveillance is a significant component of HAIs prevention because it is a means to identify outbreaks, establish endemic baseline rates of disease and evaluate control measures [57]. The first requirement for surveillance in a healthcare setting is an assessment to determine its main priorities, such as assessing the types of patients that it serves, the main medical interventions and procedures that patients have, and the types of infections patients are most vulnerable to. To choose outcomes for surveillance, possible infections, their frequency, impact and preventability should be considered in hospitals [56].

HAIs surveillance and control programmes are vital for effective IPC through activities which involve the collection and analysis of data as well as interpretation and dissemination of results. Because HAIs surveillance requires monitoring and reviewing of infection data, it enables tracking trends and identifying appropriate actions that can be taken to control and/or reduce HAIs and provides data for quality outcome indicators [58]. Nevertheless, many healthcare systems in developed and developing countries continue to use manual surveillance methods, despite the importance of HAIs surveillance programmes. The collection and analysis of patient data in real time to increase surveillance efficiency becomes challenging when automated disease surveillance technology is lacking. The use of manual surveillance resulted in inefficiencies and reporting delays, limiting the potential for effective and timely HAIs intervention [58].

When complemented with immediate feedback and efficient infection control programmes, HAIs surveillance can significantly control the prevalence of HAIs, as the results of the study on the Efficacy of Nosocomial Infection Control (SENIC) in US hospitals showed [59,60]. According to SENIC, 32% of infections that would have happened in the absence of effective infection surveillance and control systems may have been avoidable. [59,60]. The findings of this study are further supported by the findings from nosocomial infection surveillance programmes implemented across Germany that were shown to significantly reduce the rate of HAIs [61,62].

## 8. Measures Taken by the Radiology Departments to Control Infection Transmission

**Figure 1:**
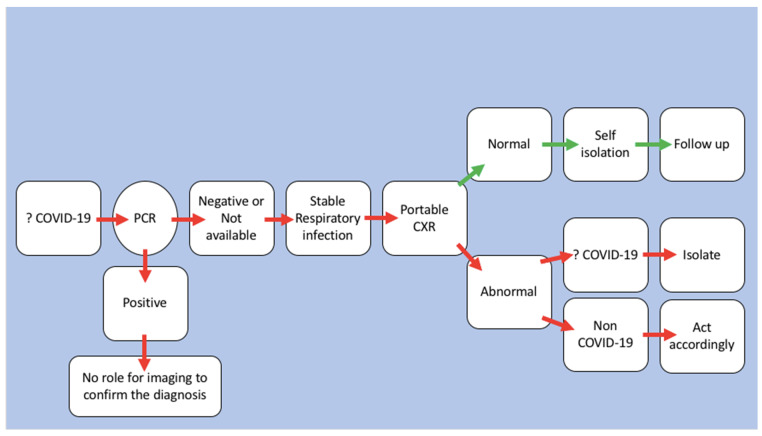
The role of imaging for patients suspected to have COVID-19 with stable respiratory infection (PCR: polymerase chain reaction, and CXR: chest X-ray).

Implementing measures to prevent infection transmission is vital in healthcare settings, particularly in radiology. Radiology departments should have plans in place to ensure that diagnostic and interventional procedures can be performed safely and without endangering patients or healthcare workers. It can be challenging, especially in an acute healthcare setting, to maintain the radiology department operating safely and with a minimal risk of HAIs. The majority of the published studies focused on the measures taken by the radiology departments in times of crises, such as the COVID-19 pandemic [17,63,64]. During the COVID-19 pandemic, the radiology departments in KSA were advised to adhere to the guidelines issued by the Saudi Arabian Ministry of Health [65]. Such guidelines followed the best international IPC practices for safe and high-quality radiology services. Figure 1 illustrates an example of a pathway for the use of imaging in individuals who are suspected to have COVID-19 with a stable respiratory infection. This pathway was created to minimize the risk of COVID-19-related HAIs. Adherence and the challenges of following this specific pathway were not addressed, prompting further research. Nonetheless, during the COVID-19 pandemic, a study conducted in KSA reported that radiology personnel were generally aware of local and international IPC rules [66].

Aside from crises, the low level of awareness and compliance with IPC standards among radiology personnel highlighted the importance of advanced training and education [16,37,54,55]. Radiology personnel should update their knowledge of IPC standards on a yearly basis, as suggested by the Occupational Safety and Health Administration and the International Society of Radiology [67,68]. The update should include a specific infection protocol for any new emergent healthcare setting, such as COVID-19. Unfortunately, guidelines alone may not be sufficient for infection control implementation and safe radiology practices. This is why multidisciplinary teams, such as hospital administrators and radiological societies, are required to reinforce these guidelines [54,69]. It is strongly advised to establish effective central coordination between the infection prevention and control department and the radiology department. Mollura et al. [70], suggested that hospital infection control personnel should play a central role in planning, practicing, and executing imaging procedures. They also should have a key role in training healthcare workers in the application of IPC standards.

Hand hygiene and PPE are two examples of basic IPC programmes that all radiology personnel should be trained on [71]. Implementing IPC standards for needle-stick injuries and keeping track of the vaccination status of radiology staff would help monitor and improve healthcare practices in ways that would help control of HAIs [24,70,72]. In the case of a needle-stick injury, it is critical for the healthcare worker to seek immediate medical attention, which should include laboratory testing for bloodborne infections [37]. However, based on the results of an online survey that examined IPC practices of interventional radiologists in the US, 25% of the respondents sustained a needle-stick injury within the previous year and only 71% of needle-stick injuries were reported [54]. Another study performed in KSA showed a similar high incidence of needle-stick injuries (22.2%), mainly among physicians, nurses, dentists, and medical technicians, with low reporting rates (46.3%) [73]. This study showed the importance of designing an educational program, providing workplace safety policies, having clear procedures for reporting needle-stick injuries and providing training for healthcare workers to avoid these injuries.

Another IPC practice specific to the radiology department is the decontamination of the imaging equipment and examination rooms. It is recommended that radiology departments contact their vendors to determine the best disinfectants for each piece of equipment and to train their responsible staff on the optimal decontamination strategies [74]. All radiology surfaces, including imaging machine table tops, should be covered with a removable sheet and changed after each patient [24]. Additionally, the radiology department should have a triage system in place as well as guidelines for recognizing suspected or infected patients, such as those with tuberculosis (TB) and MRSA [75,76]. An efficient triage system includes a complete radiology request, including the patient’s infectious status, as well as training of radiology receptionists to ask questions that will aid in the identification of such patients [75]. Tan and Kamarulzaman advocated for a separate waiting room for infected TB patients, as well as patients’ instructions to wear a surgical mask and use tissues while coughing or sneezing [75]. Additionally, they suggested surveillance of healthcare workers through the use of respiratory protection, vaccination, pre-employment testing, periodic assessment, and education. Zhang et al. [76], recommended transferring MRSA patients (with active fluid drainage) to an imaging room as soon as possible, using standard and contact precautions, and using plastic coverings or bags whenever possible to protect portable and stationary imaging equipment from contact with body fluids.

## 9. Limitations

This was a non-systematic review focused on previous non-intervention studies that examined only a few healthcare workers for IPC practices. Picton-Barnes et al. [77], conducted a systemic review of HAIs organisms in departments of medical radiation science. The small sample size of the included studies was identified as one of the limitations, making it difficult to identify the true levels of infectious organisms and limiting the applicability of the results to a broader population. In addition, the prevalence of HAIs in radiology departments may be biased, particularly in observational studies, because patients and healthcare workers can contract HAIs in other areas of the healthcare facility. Since HAIs have many aspects, it was not possible to cover them all in one paper; thus, we chose the most relevant aspects to highlight in the radiology department. In addition, the disparity in IPC practices across countries with low and high incomes was not addressed, as that was beyond the scope of this review.

## 10. Conclusions

In this review, the risk of HAIs and measures that should be taken to minimise the risk of spreading infections have been explored particularly in radiology departments sittings. As discussed, greater investments in healthcare workers’ education and training can help minimise the spread of infections and improve the success of implementing IPC strategies. Improving hand washing habits, properly using PPE, and having vaccinations against preventable diseases are crucial to protect healthcare workers and patients. The importance of having clear policies for disinfecting radiology equipment and devices and clear guides for healthcare workers to manage needle-stick injuries were discussed. Additionally, the radiology department should have a triage system in place as well as guidelines for recognizing suspected or infected patients. In conclusion, effective central coordination between the infection prevention and control department and the radiology department is essential for minimizing the transmission of infections. Investigating IPC practices in the radiology department from the perspectives of patients, hospital administrators, and healthcare workers may aid in developing a better understanding of the factors impacting IPC policy and guidelines compliance.

## Figures and Tables

**Table 1:** Common types of TBPs based on the route of transmission of the infectious agent [24,39,40,41,42,43,44].

TBPs	Mode of Transmission	Examples of Precautionary Measures
Contact	Direct or indirect contact with infected patients	▪ Wearing gloves ▪ Proper disposal of sharp items and clinical waste ▪ Minimising the number of people coming into contact with patients
Droplets	Spreading over short distance via droplets arising from the respiratory tract of one individual and travelling directly to another individual’s mucosal surface or conjunctivae	▪ Using routine environmental decontamination as sterilisation and disinfectant-based regimens ▪ Wearing eye protection ▪ Bed spacing
Airborne	Spread widely without close contact with patients via aerosols arising from the respiratory system of an infected individual and coming in contact with the mucosal surface of another individual	▪ Wearing face masks and respirators ▪ Use of Airborne Infection Isolation Rooms (AIIR) with monitored negative air pressure ▪ Provision of adequate ventilation
Enteric	Exposure to contaminated food and water as well as body secretions including vomit, and feces	▪ Hand hygiene ▪ Wearing gowns and gloves ▪ Equipment sterilisation and disinfectant-based regimens
Bloodborne	Transmitted via contamination with blood and other body secretions	▪ Hand hygiene ▪ Wearing gowns and gloves ▪ Equipment sterilisation and disinfectant-based regimens ▪ Handling sharps, needles, devices, and body fluids correctly

## Data Availability

Not applicable.
